# (*E*)-1-(Benzo[*d*][1,3]dioxol-5-yl)-3-([2,2′-bi­thio­phen]-5-yl)prop-2-en-1-one: crystal structure, UV–Vis analysis and theoretical studies of a new π-conjugated chalcone

**DOI:** 10.1107/S2056989019004912

**Published:** 2019-04-16

**Authors:** Ainizatul Husna Anizaim, Muhamad Fikri Zaini, Muhammad Adlan Laruna, Ibrahim Abdul Razak, Suhana Arshad

**Affiliations:** a X-ray Crystallography Unit, School of Physics, Universiti Sains Malaysia, 11800 USM, Penang, Malaysia

**Keywords:** crystal structure, DFT, UV–Vis, HOMO–LUMO, Hirshfeld surface

## Abstract

In the title compound, C_18_H_12_O_3_S_2_, synthesized by the Claisen–Schmidt condensation method, the essentially planar chalcone unit adopts an *s-cis* configuration with respect to the carbonyl group within the ethyl­enic bridge. In the crystal, weak C—H⋯π inter­actions connect the mol­ecules into zigzag chains along the *b*-axis direction. The mol­ecular structure was optimized geometrically using Density Functional Theory (DFT) calculations at the B3LYP/6–311 G++(d,p) basis set level and compared with the experimental values. Mol­ecular orbital calculations providing electron-density plots of HOMO and LUMO mol­ecular orbitals and mol­ecular electrostatic potentials (MEP) were also computed both with the DFT/B3LYP/6–311 G++(d,p) basis set. The experimental energy gap is 3.18 eV, whereas the theoretical HOMO–LUMO energy gap value is 2.73 eV. Hirshfeld surface analysis was used to further investigate the weak inter­actions present.

## Abstract 2

The essentially planar chalcone unit adopts an *s-cis* configuration with respect to the carbonyl group within the ethyl­enic bridge. In the crystal, weak C—H⋯π inter­actions connect the mol­ecules into zigzag chains along the *a-*axis direction.

## Chemical context   

Chalcones are organic compounds composed of open-chain flavonoids in which the two aromatic rings are joined by a three-carbon α,β-unsaturated carbonyl system (Zingales *et al.*, 2016 ▸). Compounds with the chalcone backbone are becoming important in the design of new materials, employing donor–π–acceptor (*D*–π–*A*) bridge systems to further enhance their future development for optoelectronic applications. In principle, the inter­molecular charge-transfer (ICT), HOMO–LUMO gap and optical properties can be tailored by attaching electron donors and acceptors of various electronic nature, assuring efficient *D*⋯*A* inter­actions and planarization of the entire mol­ecule (Bureš, 2014 ▸). The presence of long π-conjugated systems in chalcones have been shown to turn them into chromophores whereby certain colours can be displayed as a result of absorbing light in the visible region (Asiri *et al.*, 2017 ▸). Electron-donating and accepting groups containing these chromophores have been examined for their applications in the field of material science. Additionally, the substitution of a phenyl group into a polythio­phene compound stabilizes the conjugated π-bond system and forms a smaller band-gap material for supercapacitor applications (Mei-Rong *et al.*, 2014 ▸). As part of our ongoing studies utilizing thio­phene-ring substituents with chalcone derivatives (Zainuri *et al.*, 2017 ▸), we hereby report the synthesis, structural, UV–Vis, Hirshfeld surface and DFT analyses of the title compound, (I).
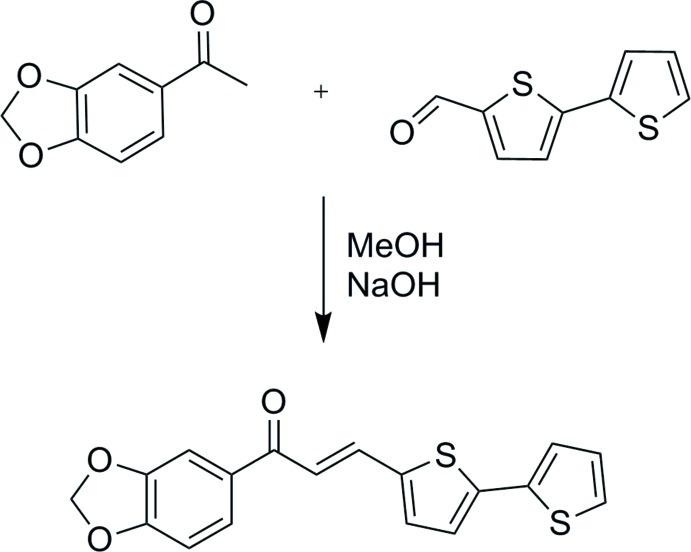


## Structural commentary   

**Figure 1:**
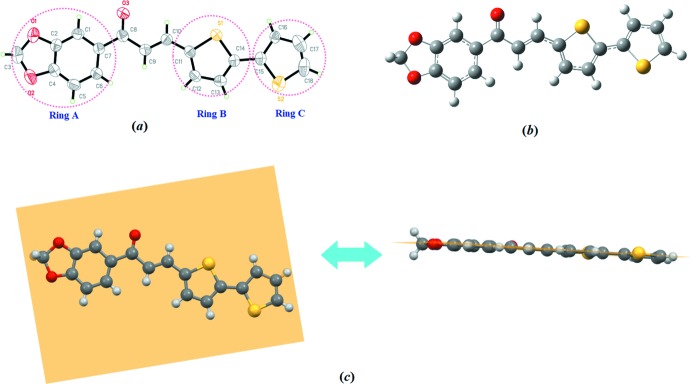
(*a*) Mol­ecular structure of the title compound showing 50% probability displacement ellipsoids; (*b*) geometry-optimized mol­ecular structure and (*c*) a representation of the mol­ecule showing the planarity of all atoms.

The experimental and optimized structures of (I) are shown in Fig. 1 ▸ *a* and 1*b*, respectively. The mol­ecular structure consists of a 1,3-benzodioxole ring system (*A*; O1/O2/C1–C7) and two thio­phene rings, *B* (S1/C11–C14) and *C* (S2/C15–C18), these substituent rings representing a donor–linker–acceptor conjugated system. Ring *A* [maximum deviation of 0.011 (4) Å at C3] forms dihedral angles of 1.88 (15) and 5.37 (16)°, respectively, with rings *B* [maximum deviations of 0.002 (3) and −0.002 (4) Å for C11 and C12, respectively] and *C* [maximum deviations of −0.009 (3) and 0.009 (3) Å for C15 and C16 respectively], respectively. The enone moiety [O3/C8–C10, maximum deviation of 0.014 (3) Å at C8] forms dihedral angles of 3.3 (2), 4.3 (2) and 7.4 (2)**°** with rings *A*, *B* and *C*, respectively. This planar conformation for the mol­ecule indicates that the 1,3-benzodioxole group and the thio­phene rings have stabilized the conjugated π-bond system.

The mol­ecule adopts an *s-cis* configuration with respect to the C8=O3 [experimental = 1.229 (4) Å and DFT = 1.227 Å] bond length within the enone moiety (O3/C8–C10). The mol­ecule is observed to be essentially planar (Fig. 1 ▸ *c*) about the C9—C10 bond with a C8—C9—C10—C11 torsion angle of 178.5 (3)**°**, whereas the corresponding DFT value is 179.6**°**. The slight difference is the result of the optimization being carried out in an isolated gaseous state whereas the experimental mol­ecular structure could easily be affected by its normal environment (Zaini *et al.*, 2018 ▸).

For the theoretical geometry optimization calculation, the starting geometries of the compound were taken from the single-crystal X-ray refinement data. The optimization of the mol­ecular geometries leading to energy minima was achieved using DFT [Becke’s non-local three parameter exchange and Lee-Yang-Parr’s correlation functional (B3LYP)] with the 6-311++G(d,p) basis set as implemented in the *Gaussian09W* software package (Frisch *et al.*, 2009 ▸). Selected bond lengths and angles for the experimental and theoretical (DFT) studies are compared in the supporting information; all values are within normal ranges (Allen *et al.*, 1987 ▸).

## Supra­molecular features   

**Figure 2:**
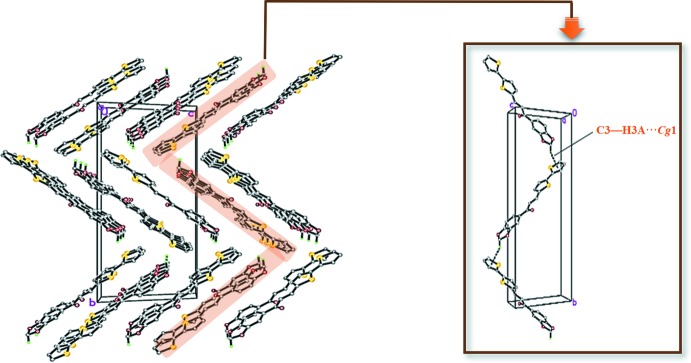
The crystal packing showing the C—H⋯π inter­actions (dashed lines).

In the crystal, mol­ecules are linked in a head-to-tail manner *via* C3—H3*A*⋯*Cg*(−*x* + 1, *y* − 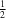, −*z* + 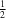) inter­actions involving ring *C* (Table 1 ▸, Fig. 2 ▸), forming zigzag chains along the *b*-axis direction. In the absence of any classical hydrogen bonds, this inter­action stabilizes the crystal structure. The chains stack along the *c*-axis direction.

## Hirshfeld surface analysis   

**Figure 3:**
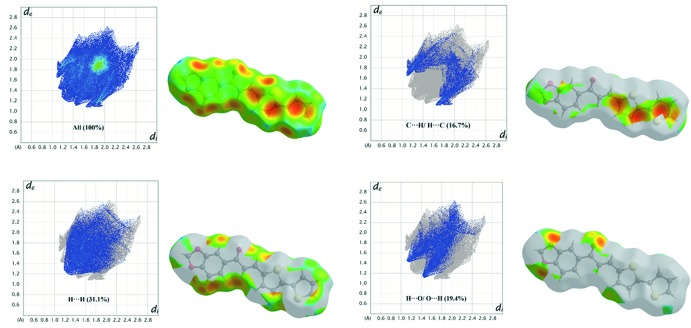
Two-dimensional fingerprint plots with a *d*_e_ view showing the percentage contributions of all inter­actions and the C⋯H/H⋯C, H⋯H and H⋯O/O⋯H inter­actions to the total Hirshfeld surface.

**Figure 4:**
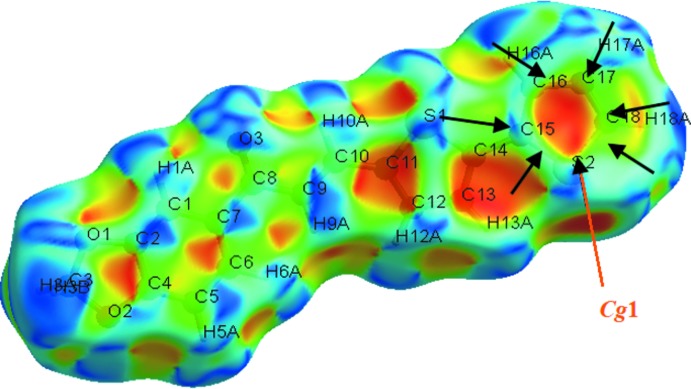
Hirshfeld surface mapped over shape-index.

Hirshfeld surface analysis was undertaken using *Crystal Explorer 3.1* (Wolff *et al.*, 2012 ▸) to investigate the mol­ecular packing. H⋯H inter­actions are the most important, contributing 31.1% to the overall crystal packing. In the fingerprint plot (Fig. 3 ▸) they are seen as widely scattered points of high density due to the large hydrogen-atom content of the mol­ecule, with *d*_e_ + *d*_i_ = 2.50 Å (*d*_i_ and *d*_e_ are the distances to the nearest atom inside and outside the surface; Shit *et al.*, 2016 ▸). C⋯H/H⋯C contacts (16.7%) are indicated by a pair of peaks at *d*_e_ + *d*_i_ = 2.75 Å, while the H⋯O/O⋯H contacts (19.4%) are represented by a pair of short spikes at *d*_e_ + *d*_i_ = 2.60 Å. The significant contributions by H⋯H, H⋯C/C⋯H and H⋯O/O⋯H inter­actions suggest that weak hydrogen bonding and van der Waals inter­actions do play relevant roles in the crystal packing (Hathwar *et al.*, 2015 ▸). The surface mapped over shape-index reveals small changes in the surface shape, indicating the C—H⋯π (Fig. 4 ▸) inter­action. The bright concave red spots in the region marked by arrows indicate atoms of the π-stacked mol­ecule, whereas the convex blue spots indicate ring atoms of the mol­ecule inside the surface (Chkirate *et al.*, 2018 ▸).

## UV–Vis and frontier mol­ecular orbital analyses   

**Figure 5:**
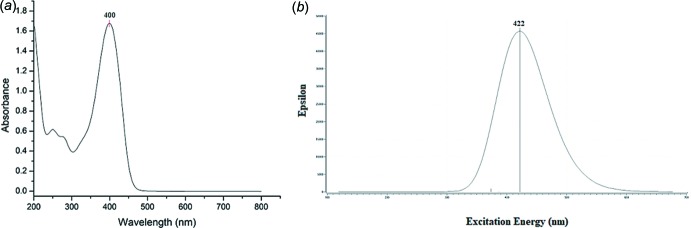
The (*a*) experimental and (*b*) calculated UV–Vis absorption spectra.

The experimental UV–Vis absorption spectrum consists of one major band that lies in the visible region at 400 nm (Fig. 5 ▸ *a*), while the simulated value is observed at 422 nm (Fig. 5 ▸ *b*). The absorption maximum is assigned to the π–π* transitions that arise from the carbonyl group (C=O) of the compound. The slight difference in wavelength is due to the fact that the experimental study is conducted in solution whereas the theoretical study is performed for a gaseous environment (Zainuri *et al.*, 2018*a* ▸). The strong cut-off wavelength for the experimental study is 455 nm (Fig. 5 ▸ *a*) with an energy band gap of 2.73 eV.

**Figure 6:**
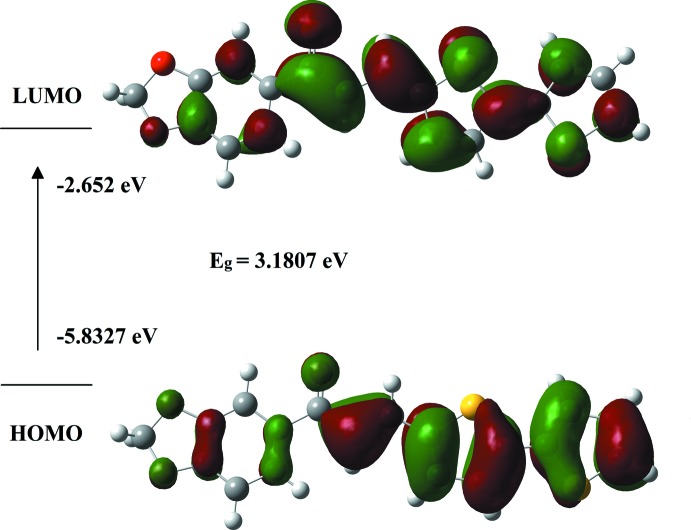
HOMO–LUMO mol­ecular orbitals showing the ground to excited state electronic transitions.

The highest occupied mol­ecular orbital (HOMO) acts as an electron donor and represents the ability to donate electrons while the lowest unoccupied mol­ecular orbital (LUMO) acts as the electron acceptor, representing the ability to accept electrons (Balasubramani *et al.*, 2018 ▸). The HOMO and LUMO electron-density plots were computed using the DFT/B3LYP/6-311 G++(d,p) basis set. The *E*_HOMO_ – *E*_LUMO_ gap is calculated to be 3.18 eV. Generally, the value of the energy gap characterizes the chemical stability of the mol­ecule (Zainuri *et al.*, 2018*b* ▸). As shown in Fig. 6 ▸, the charge densities are accumulated over the entire mol­ecule for the HOMO and LUMO states. A large HOMO–LUMO energy gap defines it as a ‘hard’ mol­ecule while a small one defines a ‘soft’ mol­ecule (Bayar *et al.*, 2018 ▸). Hard mol­ecules are less polarizable than the soft ones as there is a need of higher energy for excitation (Balasubramani *et al.*, 2018 ▸). The energy gap value in the title compound indicates good stability and a high chemical hardness.

## Mol­ecular electrostatic potentials   

**Figure 7:**
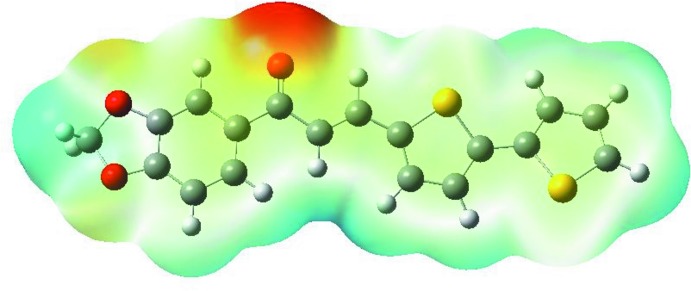
Theoretical mol­ecular electrostatic potential surface calculated at the DFT/B3LYP/6–311G++ (d,p) basis set level.

Mol­ecular electrostatic potentials (MEP) are useful in investigating the relationship between the mol­ecular structure and its physicochemical properties, visualizing the mol­ecular size and shape, along with the charge distributions in mol­ecules in terms of colour grading (Zainuri *et al.*, 2018*b* ▸). The MEP map (Fig. 7 ▸) was calculated at the B3LYP/6-311G++ (d,p) level of theory. The red- and blue-coloured regions indicate nucleophiles that are electron rich, and electrophile regions that are electron poor, respectively. The remaining white regions indicate neutral atoms. Information about inter­molecular inter­actions within the compound can be obtained from these regions (Gunasekaran *et al.*, 2008 ▸). In the title mol­ecule, the reactive site, localized in the carbonyl group, is shown in red. It possesses the most negative potential and is thus the strongest repulsion site (electrophilic attack). The blue spots indicate the strongest attraction regions, which are occupied mostly by hydrogen atoms (Zaini *et al.*, 2019 ▸).

## Database survey   

A search of the Cambridge Structural Database (Version 5.39, last update November 2017; Groom *et al.*, 2016 ▸) revealed four thio­phene-substituted compounds with a different ketone on the chalcone: (*E*)-1-(2-amino­phen­yl)-3-(thio­phen-2-yl)prop-2-en-1-one (Chantrapromma *et al.*, 2013 ▸), (2*E*)-3-(5-bromo-2-thien­yl)-1-(4-hy­droxy­phen­yl)prop-2-en-1-one (Narayana *et al.*, 2007 ▸), 1-(4-bromo­phen­yl)-3-(2-thien­yl)prop-2-en-1-one (Patil *et al.*, 2006 ▸) and (2*E*)-1-(4-bromo­phen­yl)-3-(thio­phen-2-yl)prop-2-en-1-one (Arshad *et al.*, 2017 ▸). Other related compounds that have a similar benzo[*d*]dioxol substituent on the chalcone are (2*E*)-1-(1,3-benzodioxol-5-yl)-3-(4-chloro­phen­yl)prop-2-en-1-one (Sreevidya *et al.*, 2010 ▸) and (*E*)-1-(1,3-benzodioxol-5-yl)-3-(3-bromo­phen­yl)prop-2-en-1-one (Li *et al.*, 2008 ▸).

In terms of inter­molecular inter­actions, (*E*)-1-(2-amino­phen­yl)-3-(thio­phen-2-yl)prop-2-en-1-one (Chantrapromma *et al.*, 2013 ▸) exhibits a strong inter­molecular C—H⋯O inter­action by which two adjacent mol­ecules are linked in an anti-parallel face-to-face manner into chains along the *c*-axis direction. Meanwhile, a weak inter­molecular O—H⋯O inter­action is observed in (2*E*)-3-(5-bromo-2-thien­yl)-1-(4-hy­droxy­phen­yl)prop-2-en-1-one (Narayana *et al.*, 2007 ▸). Similar to the situation in (I), weak inter­molecular C—H⋯π inter­actions link the mol­ecules of 1-(4-bromo­phen­yl)-3-(2-thien­yl)prop-2-en-1-one (Patil *et al.*, 2006 ▸) into chains along the *b*-axis direction. Lastly, an inter­molecular C—H⋯Cl inter­action, involving the terminal chloro-substituted phenyl ring, is also found in (2*E*)-1-(1,3-benzodioxol-5-yl)-3-(4-chloro­phen­yl)prop-2-en-1-one (Sreevidya *et al.*, 2010 ▸).

## Synthesis and crystallization   

A mixture of 3′,4′-(methyl­enedi­oxy)aceto­phenone (0.5 mmol) and 2,2′-bi­thio­phene-5-carboxaldehyde (0.5 mmol) was dissolved in methanol. A catalytic amount of NaOH was added dropwise with vigorous stirring. The reaction mixture was stirred for about 5 h at room temperature and then poured into ice-cold water. The resulting crude solid was collected by filtration. Single crystals were grown from an acetone solution by slow evaporation.

## Refinement   

Crystal data collection and structure refinement details are summarized in Table 2 ▸. All H atoms were positioned geometrically (C—H = 0.93 and 0.97 Å) and refined using a riding model with *U*_iso_(H) = 1.2 *U*_eq_(C). One outlier (104) was omitted from the final refinement.

## Supplementary Material

Crystal structure: contains datablock(s) I. DOI: 10.1107/S2056989019004912/jj2209sup1.cif

Structure factors: contains datablock(s) I. DOI: 10.1107/S2056989019004912/jj2209Isup2.hkl

Comparison between calculated (DFT) and X-ray of selected geometrical data. DOI: 10.1107/S2056989019004912/jj2209sup3.docx

Supporting information file. DOI: 10.1107/S2056989019004912/jj2209Isup4.cml

CCDC reference: 1899823

Additional supporting information:  crystallographic information; 3D view; checkCIF report

## Figures and Tables

**Table 1:** Hydrogen-bond geometry (Å, °) *Cg* is the centroid of the S2/C15–C18 ring.

*D*—H⋯*A*	*D*—H	H⋯*A*	*D*⋯*A*	*D*—H⋯*A*
C3—H3*A*⋯*Cg* ^i^	0.97	2.74	3.472 (5)	132

Symmetry code: (i) 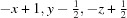.

**Table 2:** Experimental details

Crystal data	
Chemical formula	C_18_H_12_O_3_S_2_
*M*_r_	340.40
Crystal system, space group	Monoclinic, *P*2_1_/*c*
Temperature (K)	296
*a*, *b*, *c* (Å)	6.030 (1), 24.875 (5), 11.239 (2)
β (°)	114.249 (2)
*V* (Å^3^)	1537.1 (5)
*Z*	4
Radiation type	Mo *K*α
μ (mm^−1^)	0.36
Crystal size (mm)	0.19 × 0.15 × 0.06

Data collection	
Diffractometer	Bruker APEXII CCD
Absorption correction	Multi-scan (*SADABS*; Bruker, 2009 ▸)
*T*_min_, *T*_max_	0.875, 0.924
No. of measured, independent and observed [*I* > 2σ(*I*)] reflections	30820, 3024, 2096
*R*_int_	0.078
(sin θ/λ)_max_ (Å^−1^)	0.617

Refinement	
*R*[*F* ^2^ > 2σ(*F* ^2^)], *wR*(*F* ^2^), *S*	0.058, 0.164, 1.04
No. of reflections	3024
No. of parameters	208
H-atom treatment	H-atom parameters constrained
Δρ_max_, Δρ_min_ (e Å^−3^)	0.41, −0.47

Computer programs: *APEX2* and *SAINT* (Bruker, 2009 ▸), *SHELXS97* (Sheldrick, 2008 ▸), *SHELXL2014* (Sheldrick, 2015 ▸) and *SHELXTL* (Sheldrick, 2008 ▸).

## supplementary crystallographic information

### Crystal data

**Table:**

C_18_H_12_O_3_S_2_	*F*(000) = 704
*M**_r_* = 340.40	*D*_x_ = 1.471 Mg m^−^^3^
Monoclinic, *P*2_1_/*c*	Mo *K*α radiation, λ = 0.71073 Å
*a* = 6.030 (1) Å	Cell parameters from 3229 reflections
*b* = 24.875 (5) Å	θ = 2.6–19.6°
*c* = 11.239 (2) Å	µ = 0.36 mm^−^^1^
β = 114.249 (2)°	*T* = 296 K
*V* = 1537.1 (5) Å^3^	Plate, yellow
*Z* = 4	0.19 × 0.15 × 0.06 mm

### Data collection

**Table:**

Bruker APEXII CCD diffractometer	2096 reflections with *I* > 2σ(*I*)
φ and ω scans	*R*_int_ = 0.078
Absorption correction: multi-scan (SADABS; Bruker, 2009)	θ_max_ = 26.0°, θ_min_ = 2.2°
*T*_min_ = 0.875, *T*_max_ = 0.924	*h* = −7→7
30820 measured reflections	*k* = −30→30
3024 independent reflections	*l* = −13→13

### Refinement

**Table:**

Refinement on *F*^2^	0 restraints
Least-squares matrix: full	Hydrogen site location: inferred from neighbouring sites
*R*[*F*^2^ > 2σ(*F*^2^)] = 0.058	H-atom parameters constrained
*wR*(*F*^2^) = 0.164	*w* = 1/[σ^2^(*F*_o_^2^) + (0.074*P*)^2^ + 1.2156*P*] where *P* = (*F*_o_^2^ + 2*F*_c_^2^)/3
*S* = 1.04	(Δ/σ)_max_ < 0.001
3024 reflections	Δρ_max_ = 0.41 e Å^−^^3^
208 parameters	Δρ_min_ = −0.47 e Å^−^^3^

### Special details

**Table:**

Experimental. The following wavelength and cell were deduced by SADABS from the direction cosines etc. They are given here for emergency use only: CELL 0.71075 11.261 24.951 12.083 90.028 114.196 90.000
Geometry. All esds (except the esd in the dihedral angle between two l.s. planes) are estimated using the full covariance matrix. The cell esds are taken into account individually in the estimation of esds in distances, angles and torsion angles; correlations between esds in cell parameters are only used when they are defined by crystal symmetry. An approximate (isotropic) treatment of cell esds is used for estimating esds involving l.s. planes.

### Fractional atomic coordinates and isotropic or equivalent isotropic displacement parameters (Å^2^)

**Table:**

	*x*	*y*	*z*	*U*_iso_*/*U*_eq_	
S1	0.28791 (16)	0.61675 (4)	−0.12250 (9)	0.0500 (3)	
S2	0.7476 (2)	0.71610 (5)	−0.24635 (12)	0.0759 (4)	
O1	0.3608 (5)	0.35799 (12)	0.5854 (3)	0.0715 (8)	
O2	0.7813 (5)	0.35937 (12)	0.6897 (3)	0.0693 (8)	
O3	0.1080 (4)	0.48699 (11)	0.1984 (3)	0.0628 (8)	
C1	0.3315 (6)	0.42199 (14)	0.4126 (3)	0.0458 (8)	
H1A	0.1626	0.4211	0.3711	0.055*	
C2	0.4540 (6)	0.39239 (14)	0.5213 (3)	0.0468 (8)	
C3	0.5656 (8)	0.33666 (17)	0.6914 (4)	0.0681 (11)	
H3A	0.5690	0.2979	0.6837	0.082*	
H3B	0.5551	0.3452	0.7731	0.082*	
C4	0.7034 (7)	0.39318 (14)	0.5843 (3)	0.0495 (9)	
C5	0.8413 (6)	0.42354 (15)	0.5408 (4)	0.0549 (9)	
H5A	1.0100	0.4239	0.5838	0.066*	
C6	0.7201 (6)	0.45425 (14)	0.4288 (3)	0.0478 (8)	
H6A	0.8102	0.4754	0.3965	0.057*	
C7	0.4692 (6)	0.45409 (13)	0.3647 (3)	0.0388 (7)	
C8	0.3316 (6)	0.48707 (13)	0.2464 (3)	0.0439 (8)	
C9	0.4651 (6)	0.52000 (14)	0.1886 (3)	0.0472 (8)	
H9A	0.6341	0.5210	0.2290	0.057*	
C10	0.3511 (6)	0.54804 (13)	0.0810 (3)	0.0443 (8)	
H10A	0.1824	0.5455	0.0418	0.053*	
C11	0.4651 (6)	0.58250 (13)	0.0185 (3)	0.0420 (8)	
C12	0.7044 (6)	0.59448 (15)	0.0536 (3)	0.0502 (9)	
H12A	0.8296	0.5800	0.1266	0.060*	
C13	0.7433 (6)	0.63090 (15)	−0.0313 (4)	0.0514 (9)	
H13A	0.8971	0.6429	−0.0195	0.062*	
C14	0.5365 (6)	0.64701 (13)	−0.1323 (3)	0.0406 (8)	
C15	0.5055 (6)	0.68374 (13)	−0.2391 (3)	0.0440 (8)	
C16	0.2850 (6)	0.69828 (13)	−0.3490 (3)	0.0414 (8)	
H16A	0.1299	0.6861	−0.3641	0.050*	
C17	0.3416 (9)	0.73361 (17)	−0.4296 (4)	0.0707 (12)	
H17A	0.2237	0.7470	−0.5069	0.085*	
C18	0.5754 (9)	0.74663 (17)	−0.3879 (4)	0.0745 (13)	
H18A	0.6365	0.7699	−0.4319	0.089*	

### Atomic displacement parameters (Å^2^)

**Table:**

	*U*^11^	*U*^22^	*U*^33^	*U*^12^	*U*^13^	*U*^23^
S1	0.0423 (5)	0.0579 (6)	0.0427 (5)	−0.0005 (4)	0.0104 (4)	0.0114 (4)
S2	0.0742 (8)	0.0772 (8)	0.0827 (9)	−0.0029 (6)	0.0389 (7)	0.0186 (6)
O1	0.0704 (18)	0.0748 (19)	0.0736 (19)	0.0013 (15)	0.0338 (16)	0.0314 (16)
O2	0.0705 (18)	0.0690 (19)	0.0551 (17)	0.0058 (15)	0.0124 (14)	0.0231 (14)
O3	0.0410 (14)	0.0757 (19)	0.0598 (17)	−0.0031 (12)	0.0086 (12)	0.0221 (14)
C1	0.0394 (18)	0.050 (2)	0.046 (2)	0.0012 (15)	0.0160 (15)	0.0002 (16)
C2	0.053 (2)	0.043 (2)	0.048 (2)	−0.0008 (16)	0.0243 (17)	0.0030 (16)
C3	0.094 (3)	0.057 (3)	0.054 (2)	0.003 (2)	0.031 (2)	0.011 (2)
C4	0.058 (2)	0.044 (2)	0.0394 (19)	0.0029 (17)	0.0126 (17)	0.0026 (15)
C5	0.0418 (19)	0.058 (2)	0.053 (2)	−0.0009 (17)	0.0081 (17)	0.0032 (19)
C6	0.0446 (19)	0.049 (2)	0.046 (2)	−0.0024 (15)	0.0139 (16)	0.0039 (16)
C7	0.0427 (17)	0.0354 (17)	0.0361 (17)	−0.0030 (14)	0.0139 (14)	−0.0039 (14)
C8	0.0471 (19)	0.0412 (19)	0.0389 (18)	−0.0015 (15)	0.0131 (15)	−0.0026 (15)
C9	0.0430 (18)	0.049 (2)	0.046 (2)	−0.0044 (15)	0.0148 (16)	0.0025 (17)
C10	0.0440 (18)	0.0462 (19)	0.0408 (19)	−0.0014 (15)	0.0155 (15)	0.0005 (16)
C11	0.0443 (18)	0.0437 (19)	0.0364 (18)	0.0052 (14)	0.0152 (15)	0.0000 (15)
C12	0.0418 (19)	0.061 (2)	0.042 (2)	0.0075 (16)	0.0112 (15)	0.0116 (17)
C13	0.0394 (18)	0.059 (2)	0.055 (2)	0.0030 (16)	0.0191 (17)	0.0067 (18)
C14	0.0456 (18)	0.0368 (18)	0.0411 (18)	0.0019 (14)	0.0196 (15)	−0.0016 (14)
C15	0.056 (2)	0.0369 (18)	0.0430 (19)	0.0032 (15)	0.0236 (16)	−0.0011 (15)
C16	0.0505 (19)	0.0413 (19)	0.0311 (17)	−0.0042 (15)	0.0155 (15)	0.0005 (14)
C17	0.091 (3)	0.067 (3)	0.048 (2)	0.024 (2)	0.021 (2)	0.006 (2)
C18	0.115 (4)	0.059 (3)	0.066 (3)	0.004 (3)	0.055 (3)	0.014 (2)

### Geometric parameters (Å, º)

**Table:**

S1—C14	1.720 (3)	C6—H6A	0.9300
S1—C11	1.728 (3)	C7—C8	1.491 (4)
S2—C18	1.682 (4)	C8—C9	1.473 (5)
S2—C15	1.698 (3)	C9—C10	1.318 (5)
O1—C2	1.378 (4)	C9—H9A	0.9300
O1—C3	1.420 (5)	C10—C11	1.448 (4)
O2—C4	1.369 (4)	C10—H10A	0.9300
O2—C3	1.425 (5)	C11—C12	1.364 (5)
O3—C8	1.229 (4)	C12—C13	1.404 (5)
C1—C2	1.356 (5)	C12—H12A	0.9300
C1—C7	1.409 (5)	C13—C14	1.357 (5)
C1—H1A	0.9300	C13—H13A	0.9300
C2—C4	1.375 (5)	C14—C15	1.458 (5)
C3—H3A	0.9700	C15—C16	1.441 (5)
C3—H3B	0.9700	C16—C17	1.401 (5)
C4—C5	1.355 (5)	C16—H16A	0.9300
C5—C6	1.395 (5)	C17—C18	1.330 (6)
C5—H5A	0.9300	C17—H17A	0.9300
C6—C7	1.383 (4)	C18—H18A	0.9300
			
C14—S1—C11	92.72 (16)	C10—C9—C8	121.6 (3)
C18—S2—C15	92.9 (2)	C10—C9—H9A	119.2
C2—O1—C3	105.6 (3)	C8—C9—H9A	119.2
C4—O2—C3	105.3 (3)	C9—C10—C11	125.8 (3)
C2—C1—C7	117.6 (3)	C9—C10—H10A	117.1
C2—C1—H1A	121.2	C11—C10—H10A	117.1
C7—C1—H1A	121.2	C12—C11—C10	130.2 (3)
C1—C2—C4	122.1 (3)	C12—C11—S1	109.9 (3)
C1—C2—O1	128.3 (3)	C10—C11—S1	119.9 (2)
C4—C2—O1	109.6 (3)	C11—C12—C13	113.4 (3)
O1—C3—O2	109.0 (3)	C11—C12—H12A	123.3
O1—C3—H3A	109.9	C13—C12—H12A	123.3
O2—C3—H3A	109.9	C14—C13—C12	113.9 (3)
O1—C3—H3B	109.9	C14—C13—H13A	123.0
O2—C3—H3B	109.9	C12—C13—H13A	123.0
H3A—C3—H3B	108.3	C13—C14—C15	129.5 (3)
C5—C4—O2	127.7 (3)	C13—C14—S1	110.1 (3)
C5—C4—C2	121.7 (3)	C15—C14—S1	120.4 (3)
O2—C4—C2	110.5 (3)	C16—C15—C14	128.6 (3)
C4—C5—C6	117.3 (3)	C16—C15—S2	110.4 (2)
C4—C5—H5A	121.3	C14—C15—S2	121.0 (3)
C6—C5—H5A	121.3	C17—C16—C15	109.2 (3)
C7—C6—C5	121.6 (3)	C17—C16—H16A	125.4
C7—C6—H6A	119.2	C15—C16—H16A	125.4
C5—C6—H6A	119.2	C18—C17—C16	115.4 (4)
C6—C7—C1	119.6 (3)	C18—C17—H17A	122.3
C6—C7—C8	123.5 (3)	C16—C17—H17A	122.3
C1—C7—C8	116.9 (3)	C17—C18—S2	112.1 (3)
O3—C8—C9	120.5 (3)	C17—C18—H18A	123.9
O3—C8—C7	119.9 (3)	S2—C18—H18A	123.9
C9—C8—C7	119.6 (3)		
			
C7—C1—C2—C4	0.2 (5)	C7—C8—C9—C10	177.2 (3)
C7—C1—C2—O1	−179.5 (3)	C8—C9—C10—C11	178.5 (3)
C3—O1—C2—C1	179.0 (4)	C9—C10—C11—C12	−0.8 (6)
C3—O1—C2—C4	−0.6 (4)	C9—C10—C11—S1	−179.8 (3)
C2—O1—C3—O2	0.5 (4)	C14—S1—C11—C12	−0.3 (3)
C4—O2—C3—O1	−0.2 (4)	C14—S1—C11—C10	178.8 (3)
C3—O2—C4—C5	−179.0 (4)	C10—C11—C12—C13	−178.6 (3)
C3—O2—C4—C2	−0.2 (4)	S1—C11—C12—C13	0.4 (4)
C1—C2—C4—C5	−0.2 (6)	C11—C12—C13—C14	−0.3 (5)
O1—C2—C4—C5	179.5 (3)	C12—C13—C14—C15	−179.5 (3)
C1—C2—C4—O2	−179.2 (3)	C12—C13—C14—S1	0.1 (4)
O1—C2—C4—O2	0.5 (4)	C11—S1—C14—C13	0.1 (3)
O2—C4—C5—C6	178.7 (3)	C11—S1—C14—C15	179.8 (3)
C2—C4—C5—C6	0.0 (6)	C13—C14—C15—C16	176.6 (3)
C4—C5—C6—C7	0.3 (6)	S1—C14—C15—C16	−3.0 (5)
C5—C6—C7—C1	−0.4 (5)	C13—C14—C15—S2	−3.2 (5)
C5—C6—C7—C8	178.7 (3)	S1—C14—C15—S2	177.20 (19)
C2—C1—C7—C6	0.1 (5)	C18—S2—C15—C16	−1.2 (3)
C2—C1—C7—C8	−179.0 (3)	C18—S2—C15—C14	178.6 (3)
C6—C7—C8—O3	−176.3 (3)	C14—C15—C16—C17	−178.2 (3)
C1—C7—C8—O3	2.9 (5)	S2—C15—C16—C17	1.6 (4)
C6—C7—C8—C9	3.1 (5)	C15—C16—C17—C18	−1.4 (5)
C1—C7—C8—C9	−177.8 (3)	C16—C17—C18—S2	0.5 (5)
O3—C8—C9—C10	−3.5 (5)	C15—S2—C18—C17	0.4 (4)

### Hydrogen-bond geometry (Å, º)

*Cg* is the centroid of the S2/C15–C18 ring.

**Table:**

*D*—H···*A*	*D*—H	H···*A*	*D*···*A*	*D*—H···*A*
C3—H3*A*···*Cg*^i^	0.97	2.74	3.472 (5)	132

Symmetry code: (i) −*x*+1, *y*−1/2, −*z*+1/2.
