# Supplementary material for: (E)-1-(Benzo[d][1,3]dioxol-5-yl)-3-([2,2′-bi­thio­phen]-5-yl)prop-2-en-1-one: crystal structure, UV–Vis analysis and theoretical studies of a new π-conjugated chalcone
Source: Acta Crystallogr E Crystallogr Commun. 2019 Apr 16;75(Pt 5):632–7. doi: 10.1107/S2056989019004912 (PMC6505620; doi:10.1107/S2056989019004912)
Supplement: Supplementary file 3 [file e-75-00632-sup3.docx]

**Supplementary Material**

**Table S1** Comparison between calculated (DFT) and X-ray of selected geometrical data for **I**.

| Geometric parameters | Experimental Value (Å), (°) | DFT Value (Å, °) |
| --- | --- | --- |
| Bond lengths  O3—C8  C7—C8  C8—C9  C9—C10  C10—C11  C14—C15  C15—C16  O1—C2  O1—C3  O2—C4  O2—C3  S1—C14  S1—C11  S2—C18  S2—C15 | 1.229 (4)  1.491 (4)  1.473 (5)  1.318 (5)  1.448 (4)  1.458 (5)  1.441 (5)  1.378 (4)  1.420 (5)  1.369 (4)  1.425 (5)  1.720 (3)  1.728 (3)  1.682 (4)  1.698 (3) | 1.227  1.500  1.480  1.350  1.439  1.447  1.379  1.373  1.433  1.366  1.438  1.744  1.755  1.730  1.753 |
| Bond angles  C12—C11—C10  C10—C11—S1  C9—C10—C11  C10—C9—C8  O3—C8—C9  O3—C8—C7  C9—C8—C7  C1—C7—C8  C6—C7—C8 | 130.2 (3)  119.9 (2)  125.8 (3)  121.6 (3)  120.5 (3)  119.9 (3)  119.6 (3)  116.9 (3)  123.5 (3) | 130.9  119.3  126.7  120.0  120.8  119.7  119.4  116.4  123.8 |
| Torsion angle  C1—C7—C8—C9  O3—C8—C9—C10  C9—C10—C11—S1  C9—C10—C11—C12 | -177.8 (3)  -3.5 (5)  -179.8 (3)  -0.8 (6) | -172.2  0.5  -179.9  0.04 |
